# Social Community Teams’ Creation of Service Integration Through Boundary Work and Play with Their Stakeholders

**DOI:** 10.5334/ijic.7624

**Published:** 2024-07-01

**Authors:** Martian Slagter, Marjolein Van Offenbeek, Manda Broekhuis

**Affiliations:** 1 University of Groningen, NL

**Keywords:** service integration, social community teams, team boundary work, team boundary play, stakeholders

## Abstract

In many European countries, responsibilities for social care have been shifted to municipalities to enhance accessibility and stimulate integration of care and social services, and to cut costs. Multidisciplinary local Social Community Teams (SCTs) have become increasingly responsible for the provision of these integrated services, requiring them to collaborate with local health and societal organisations. To collaborate and to integrate services the SCTs must work across their own and stakeholders’ boundaries (e.g., domain specific boundaries). We investigated how boundary work in SCTs’ practices contributes to service integration in a dynamic multi-stakeholder context. In our embedded case study, for 18 months, we followed three SCTs in their efforts to integrate services, and used data from multiple sources, including bi-weekly questionnaires in which SCT members reflect on their stakeholder-directed goal achievements.

The case analysis yielded four takeaways. First, it demonstrates how SCTs’ bottom-up formulation of a long-term service integration vision brought internal coherence (boundary reinforcement), while the short-term action-goals increased collaboration with stakeholders (boundary spanning). Second, only SCTs that managed to incorporate constraints into their action-goals and practices, and to span and play with boundaries, continued with integrating services just-by-doing. Third, two stakeholder characteristics facilitated the SCTs’ boundary spanning: well-organized stakeholders and prior familiarity with the stakeholder. Fourth, a new boundary work type emerged, “boundary play”, consisting of informal, experimental collaboration efforts with stakeholders contributing to emergent service integration.

## Introduction

Living in safe and supportive communities is highly valued to enhance social health and well-being [1]. Since 2015 in the Netherlands, social care responsibilities shifted from the national level to the municipalities to improve social care’s accessibility, to integrate health and care services into citizens’ local environment and to cut costs [2]. Now 80% of 345 municipalities work with Social Community Teams (SCTs) that deliver a broad spectrum of social support for citizens’ increasingly complex daily life issues [3,4]. The integration into the local environment requires SCTs to collaborate with a wide range of public and private actors in the social domain, such as Safe at Home, housing corporations and general practitioners. The complexity and interwovenness of the problems citizens experience (e.g., psychiatric, debts, child abuse) requires service integration [3]. However, stakeholder collaboration is hindered by differences in and unclarities about accountability, financing, information exchange policies, rationing criteria, or stakeholders working at cross-ends [3,5,6]. Practitioners’ consecutive yet fragmented initiatives and the growing scholarly attention for these, underline the relevance to improve interorganisational collaboration for service integration in the Netherlands and beyond [e.g., 7,8,9,10,11,12]. In this collaboration SCTs’ boundaries emerge. They need to exchange not only expertise and information with stakeholders across boundaries, but their collaboration in the actual delivery of services also requires reconsidering and possibly revising their own boundaries. For example, who will invest time in the support, and who can be held accountable for what in the joint treatment-plan, or who needs to be informed when a client deteriorates?

SCTs need to devise ways to simultaneously collaborate with external stakeholders in delivering integrated services to their joint client group, and maintain a recognisable identity for these clients, and their financers. A focus on their boundary work with these stakeholders may therefore unravel how SCTs achieve integrated services with other caretakers or organisations.

Our research question is: “*how boundary work in SCTs’ practices contributes to service integration in a dynamic multi-stakeholder context*”. We observed and analysed three SCTs’ conscious integration efforts for an 18-month period. We explored their collaboration and experimentation with their stakeholders. With this qualitative embedded case study, we contribute to the practices of the social community teams by a theory-informed, bottom-up, incremental approach for service integration [13,14].

## Theoretical framework

Team boundary work has been widely studied by scholars [15,16,17,18,19]. We define a boundary as a domain of interactions of a team with stakeholders in its environment to build, protect or change practices [17]. Thereby, we define a stakeholder as any group or individual who can affect or is affected by the achievement of the teams’ objectives [20]. A boundary provides a team a source for innovation with stakeholders, as well as a buffer for the competing external demands to protect a teams’ resources and identity [15]. Experimenting with stakeholders to develop integrated services means creating clarity about teams’ and stakeholders’ boundaries: Who is doing what, how and why and what will we be doing together?

In addition, we define team boundary work as: a purposeful team effort to influence boundaries affecting the team, the organisation, and the stakeholders [18]. Boundary work occurs when a team collaborates with internal (stakeholders belonging to the same organisation) and external stakeholders to achieve a team’s goals. These actions unfold within and across a team entailing inclusion or exclusion processes with stakeholders. For example: who belongs to a specific team or who is allowed to do which activities, who has access to certain knowledge, and which activities are we conducting together. Team boundary work can be different in focus and purpose. Teams’ activities that show an inward focus address issues such as ‘what do we want to achieve and what is our role’? To develop, build or clarify the boundary they develop boundary reinforcing activities [15,16,19]. Other boundary work shows an outward focus as a reaction to the external world or to reach out and collaborate with others. To protect, delineate or close the boundary, teams engage boundary buffering activities in reaction to threats or competing demands [21,22]. For example, teams need to protect time and other resources to execute their primary tasks effectively. Finally, to collaborate, exchange knowledge and obtain resources, teams and stakeholders create new joint boundaries serving shared goals and interests and develop boundary spanning activities [19].

In health and social care research, the team boundary work lens offers a perspective to explore the way teams work on their boundaries to achieve service integration with stakeholders [22,23].

## Methodology

### Research design

In this research, we followed three social community teams (SCTs) from one large social care organisation in the Netherlands, who had dedicated themselves to improve service integration. We followed them for 18 months, after their management had invited them to develop more collaborative practices with stakeholders, while remaining separate teams representing their own organisation. To secure processual progress and scientific distance, the researchers created two roles. The second author acted as process facilitator in quarterly meetings, contributing to discussions, providing information, and supporting the SCTs in their project. The first author took an observing role during sessions, collected the data, and took the lead in analysing the data [24].

### Case setting and selection

The sampled SCTs’ generalist role was to support the municipality’s youth and their caretakers with a broad spectrum of primary social care services, each SCT focusing on a different place where youth spend time during their daily life [25] (Table 1).

**Table 1:** The sampled teams.


SCT’S NAME	SCT’S FOCUS	COMPOSITION	STC’S STAKEHOLDERS

School (SCH)	Focuses on vocational schools in the city. Supports students (age 16–24 year) to find and keep suitable housing, since having a safe living place is a critical factor in preventing school dropout.	1 Coordinator 1 Behavioral expert 1 Expert Social Support Act (SSA)	Municipal team school Students Housing corporations Vocational schools Colleague municipal and provincial SCTs Organisations offering ambulatory support (OAS) Municipality (housing and social support act experts)

Sport (SPO)	Focuses on sport clubs in the city. Aims to support young football club members, as well as non-members and their caretakers. In this setting young people likely develop social skills and a healthy lifestyle as a supporting condition for social health and well-being.	1 Coordinator 3 Social workers	SCT Sport Caretakers Colleague SCTs Management Team Sport club: trainers, and board members Health and care organisations Government, rules, and procedures

Street (STR)	Focuses on a multi-problem street in a neighbourhood. Here, the SCT tries to strengthen young adults’ social networks and to learn about what is happening on the street and in people’s home.	1 Coordinator/social worker 2 Youth experts 1 Social worker 1 Expert SSA	SCT Street Inhabitants Colleague SCTs Management Team Municipal area team, other organisations Health and care organisations Government, rules, and procedures


**Figure 1:**
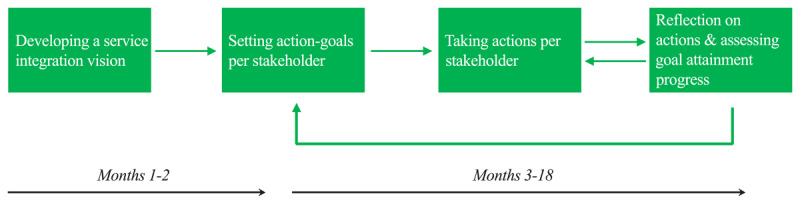
The SCTs’ bottom-up, incremental service integration approach.

The first two months, each SCT articulated their own challenging service integration vision and identified the stakeholders for realising it. Then they were individually invited to set their own action-goal per stakeholder to enhance their motivation and create room for experimenting [26]. The following months, they worked on these and discussed their progress, fuelled by a bi-weekly self-assessment of and reflection on their goal attainment (Figure 1).

### Data collection

We collected data from multiple sources. First, team members’ biweekly self-assessment of their progress and reflections were collected through a digital questionnaire (QUEST). The questionnaires served to support the SCTs in cyclically developing and reflecting on short-term actions towards achieving self-set service integration goals, and generated research data (Table 2).

**Table 2:** Self-assessment format.


BIWEEKLY QUESTIONNAIRE: QUESTIONS AND SELF-ASSESSMENT SCORES						

Per stakeholder-related goal	Describe your activities and their effect on this goal:					

	To what extent did you succeed in your goal (X) for stakeholder (Y)?					

*Six-point Likert Scale*	*–1*	*0*	*1*	*2*	*3*	*4*

	*Further away from the goal*	*Nothing has changed*	*A small part of the goal has been achieved*	*Much of the goal has been achieved*	*The goal has been achieved*	*More has been achieved than the set goal*


We used a WhatsApp group for reminders. No questionnaires were sent during participants’ holidays. STR asked to switch to monthly questionnaires during the last six months, allowing them more time to develop actions. A second source comprised the transcripts of eight SCT meetings (TM; 8, 75 hrs of recordings) and two focus group interviews (FG; 2, 6 hrs. of recordings). Additionally, we collected field notes (NOTE) of 20 SCT meetings, emails, and WhatsApp group communications. Finally, to contextualise this data [18], we conducted and transcribed six internal and seven external stakeholder interviews (12 hrs. of recordings).

### Data analysis

We coded the cumulating data in three rounds, using Atlas ti to keep track of the increasing number of documents and of the coding process. As incomplete questionnaires were not included, the final analysis was based on 205 questionnaires and 69 TM-, FG-, NOTE- documents. First, we inductively coded the data that were collected after six months. This resulted in a preliminary in vivo coding of the SCTs’ collaborative activities within the team and with stakeholders [27]. In the same month, we visualised the first coding in a mind map, which we shared with the SCTs to better interpret their activities and reflect on their progress. In the second round, we categorised the inductive codes using boundary work concepts as sensitising theory for second-order coding [13,28]. We again presented this descriptive analysis of the SCTs goal setting, boundary work and service integration attempts after nine months with the SCTs and managers. The two feedback rounds also helped our interpretation of the data, and we included the meeting transcripts in the subsequent coding rounds. In the third coding round, we continued the second order coding by merging action verbs in preliminary aggregated themes of boundary work and service integration achievements. We added TM-, FG- and NOTE documents when they became available after meetings. The last analysis stage took place after the 18th months. We looked for patterns between the boundary work types of each SCT and their integrated services efforts.

## Findings

**Figure 2:**
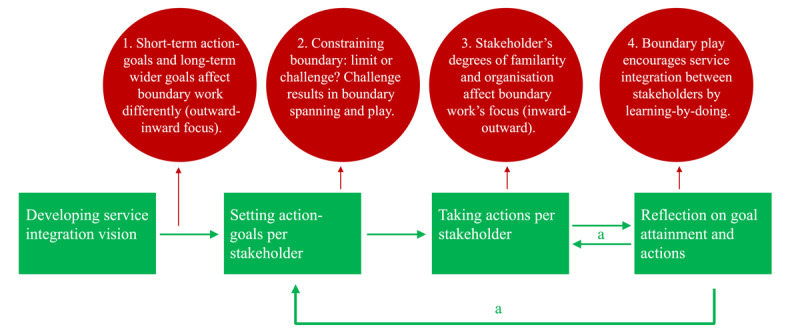
Patterns in the SCTs’ bottom-up, incremental service integration approach. a = Reflecting on and updating the action-goals.

We found five team boundary work types that all SCTs engaged in (Table 3), but in varying sequences and to different extents leading to differences in the SCTs’ service integration achievements (Table 4). We identified four main patterns in the SCTs’ boundary work that in combination may explain the different outcomes of their service integration efforts (Figure 2). One common finding was that teams reported little boundary buffering activities such as priority setting and declining managerial demands, which seemed to accord with their continuing struggling with the available time to develop integrated services as demonstrated in the following quote:

“It should be four hours on paper, but it doesn’t feel like that (……), the pressure increases because the [regular] caseload is increasing because of other things. And for me, I was asked to do things for other neighbourhoods. So, this [actions for or with stakeholders] fades away…” (NOTE20211014 STR).

**Table 3:** SCTs Team boundary work types.


AGGREGATED THEME	DESCRIPTION AND FOCUS	2nd ORDER CODES	EXAMPLE QUOTES

**A. Building and reinforcing boundary** [9]	Inward focus: the team’s activities to clarify issues and actions and build a foundation to reach out to the targeted stakeholders.	Discuss and improve actions.	“We scheduled a repeating meeting about this project where we simultaneously fill out the questionnaire. In this meeting we share our visions and experiences as well.” (QUEST21-11 SPO-1) “We’ve assessed the housing project and found it to be running smoothly with clear internal processes. However, we’re now exploring possibilities for further pioneering. Despite the successes, it’s evident there are ongoing housing challenges for vocational students, though these fall outside the project’s scope.” (QUEST21-23 SCH-2) “In our team’s collaboration, we have set new goals that resonate with all of us. You can feel that we are all on the same page and know exactly what we are doing together.” (QUEST21-29 STR-6)

		Learn from actions, reflect in the team.	“I consider us as a real team. We discuss progress, and learn not only from cases, but also how this project relates to other important areas for our organisation.” (QUEST21-23 SPO-3) “During our actions’ evaluation, we all came up with points on how to approach this topic even more effectively. For example, conducting phone calls with housing corporations and holding case discussions about students’ housing issues within the team.” (QUEST21-11 SCH-1)

**B. Boundary buffering** [9]	Outward focus: the team’s activities to protect the boundary against disturbances from outside the team.	Discuss threats to achieve goals.	“To which extent the management supports this project, I don’t know. I get the impression that there are many, and many different projects with divergent themes (…) I miss consistency. I would like to set up a tailoring session for our projects.” (21-17 SPO-3) “Currently, issues like illness, growing waiting lists, and heavier caseloads impacting some team members’ levels of engagement. I’ve addressed these concerns, prompting action from the management team. We’re now receiving temporary support from youth colleagues, allowing us to refocus on working and thinking differently and take more time to reflect.” (QUEST21-6 STR-2)

**C. Boundary spanning** [9; 15]**: inside out**	Outward focus, the team’s actions aiming to inform others and convey ideas, and services (sending).	Inform and inspire others.	“At school, last week, I discussed with the mentors about housing and how to apply [this flowchart] to the students for our programme. Often, I notice that people don’t know much about this topic, so very important, perhaps it is an idea to visit the teacher teams to inform them about our flowchart.” (QUEST21-7 SCH-1) “Our approach in the street is well presented to the management team. Our manager knows well and propagates the importance of this approach to other colleagues.” (QUEST21-3 STR-3)

**D. Boundary spanning** [9; 15]**: outside in**	Outward focus: the team’s actions to obtain new insights, information or means, enabling the team to negotiate and collaborate with the targeted stakeholder (receiving).	Obtain information and support.	“I spoke with the manager about the project. Later, she called me back and pointed out the possibility to apply for a subsidy for projects addressing ‘the impact of COVID-19 on youth’. We have added it to our team’s action list.” (QUEST21-19 SPO-1) “Within our team, we built on the ground-breaking professional and the housing topic as well. I am designing the ‘flowchart housing’ together with a colleague. We need input from other colleagues, and we talked about this extensively. I talked to colleagues about enrolling for the housing project and the expected changes as well.” (QUEST21-7 SCH-1) “I only heard about the area restriction [from inhabitant] from the police, but not officially. The municipality issued this restriction, not the police. I’ve reached out and expressed our desire to be involved […].” (STR-NOTE21-1202)

**E. Boundary play** [29]	Outward focus: the team’s actions to blur the boundaries, experiment with others, and learn by doing iteratively.	Try something new.	“In our conversations, the club mentioned their concerns about the youngsters but felt no possibilities to do more about it. Then I thought: ‘This is where we can join in’ All we must do now is to communicate clearly, and especially experiment together.” (21-11 SPO-3) “Past two weeks, we have been working on clarifying our goals for making them actionable. Additionally, we supported the early detection [of housing problems] in the teachers’ teams, that is important. This involves breaking routines and trying something new […].” (SCH-TM20210330)

		Learn together.	“We scheduled a monthly meeting with the club. There, we discuss running cases in which we and the board consider things from our different perspectives.” (QUEST21-11 SPO-3) “The cards are designed [with three questions to discover housing issues]. Now just printing remains. I noticed that asking about housing is now integrated in the intake process […].” (QUEST22-7 SCH-1)


**Table 4:** SCTs’ Service integration.


AGGREGATED THEME	DESCRIPTION	2nd ORDER CODES	EXAMPLE QUOTES

**Service integration**	The SCT’s description of the achieved service integration with one or more stakeholders.	An observed need for	“Due to neighbourhood disturbances, an inhabitant moved to another area last year for safety reasons, with the approval of involved authorities. Now, a different authority wants to move the person back to the street, prioritising the person’s wishes over neighbourhood safety. We need to organise a meeting to discuss the impact on the whole street and learn how to operate in an integrated way.” (NOTE20210303STR)

		As a failed or not yet realised outcome	“I referred in an intake conversation with a student with problems (including housing) to social work. Therefore, the student had to wait longer despite the urgency. This makes me dependant on the other SCT and reduces my ability to support the non-self-reliant student. I’m concerned about these situations” (QUEST21-8 SCH-3)

		As a realised outcome	“It is about identifying, together with the trainers, youth in difficult circumstances and, using all the signals from referees and trainers. Then we can apply for assistance for acquiring some sportwear, with the support of another SCT as well, and the kid can continue to play.” (NOTE 210507SPO)


The four main patterns presented below, need to be interpreted against this context of experienced resource constraints.

### Short-term action-goals increase boundary spanning; long-term wider goals strengthen boundary reinforcement

Short-term action-goals directed SPO and SCH to reach out to their external stakeholders and to set up joint activities (boundary spanning) (Table 3:D). SPO focused on the sport club and the sporters’ caretakers and SCH on a housing corporation and an organisation focused on housing for vocational students for actions. Their goal achievement evaluations led to further short-term actions as planning new meetings or agreements about joint activities which empowered them to experiment with the stakeholder (Table 3:E). Next quote illustrates a joint effort between SPO and the sports club.

“We discussed which specific action-goals we wanted to set for this project and for which topics we are going to act. We agreed about how we are going to achieve the action-goals.” (QUEST21-6 SPO-2)

While all SCTs discussed about what they encountered during work, only in combination with short-term action-goals and their evaluations, the discussions led to new actions and collaboration with the stakeholder talking about who is doing what the coming period. For example, who is involving the trainers (SPO) or what could be a good follow up action for enhancing the awareness of the effect of housing problems for students (SCH).

The wider goals like “discuss our approach with colleagues when possible”, induced more inward focused team dynamics. STR discussed its approach and how to manage their role differences (Table 3:A). These discussions about what they wanted to achieve for the street in the long run enhanced their ideas about their service integration approach towards the inhabitants but did not stimulate STR to step into concrete actions. At this point, they did not feel sufficient progress yet. The next quote illustrates their awareness about this:

“Last week we concluded that our current goals are too far away, too wide, and we cannot feel them yet.” (QUEST21-6 STR-2)

Such internal team discussions seemed to result in stronger role and competency clarity, which the team experienced as a step forward to work on joint goals with others.

“You really notice the difference in approaches. As a caregiver, I am ingrained to observe or remain silent about another family, you know. Essentially, I always came into the picture as a caregiver when there were issues. [….] Now that I am working with a social worker in the neighbourhood, we have interesting conversations about it.” (QUEST21-11 STR-3)

This progress accelerated the development and distribution of the flyer for street inhabitants and the organisation and execution of a webinar for colleagues to communicate about their approach for the street.

When the short-term action-goals overlapped the SCTs’ broader vision, we noticed a tendency to prioritise immediate regular tasks and cases, which ended up affecting the inspiration and motivation negatively, rather than enhancing their focus on service integration as intended.

### Different boundary work in reaction to similar constraining boundaries

STCs responded differently to similar constraining boundaries, which we illustrate by the following two examples. First, the boundary of the General Data Protection Regulation act (GDPR), an obligatory guideline of the European Union to safeguard data and privacy of individuals, applying to the three SCTs equally. STR felt very restricted by this act to meet inhabitants in the street and to actively create a mutual understanding for each other, or to collaborate with other care institutions.

“We struggle with numerous problems that we run into, such as the GDPR. What can we share, and which information remains invisible because [external stakeholder] is involved. Via our conversations [in team] about it we obtained more clarity.” (QUEST21-11 STR-3)

While SPO and SCH were aware of the requirements to comply with the GDPR, they included these regulations into their action-goalsetting by redefining them as a challenge and made this a topic in their mutual conversations. This ensured SPO and SCH to continue their collaboration with the external stakeholders, enfolding boundary spanning or boundary play (Table 3:C,D,E) as the following quote in a mail to others shows.

“So far, [what we have accomplished] fully aligned with our vision for this project, but I am considering the privacy part. I shared a signal without consent. [….] In the future, how do we act properly? In addition, this caretaker cannot read or write.” (NOTE20210930 SPO).

Second, the SCTs had to cope with the COVID-19 measures of the Dutch government applying for all citizens in the country, for example a lock down period. Initially, this prevented the SCTs from approaching their clients and other stakeholders, which impaired the action-goal’s progress resulting in a dip in their progress evaluations. Team STR could not go out on the street to connect with the inhabitants to make themselves known. Instead, they first focused on its internal discussion about its goals for the street and other stakeholders (Table 3:A) before switching to the actual reaching out and walking around in the street, or to convey their ideas to others (Table 3:C). In contrast, SPO and SCH continued their collaboration with the sports clubs and schools by using diverse communication channels, such as mail or videocalls. Consequently, SPO and SCH reported goal attainment progress, underpinned by the following quotes:

“I talked [with stakeholder] about the importance of early signalling and the way we are working on it. This is quite a challenge in Covid times, but especially now we must be extra vigilant.” (QUEST20-47 SCH-1)

“Soon we will publish a text about our whereabouts for the website of the club so parents will be informed, and they can use our services when necessary. For now, this is an important step forward because we cannot visit the club to get more recognisability.” (QUEST21-7 SPO-2)

### Stakeholder’s degrees of familiarity and organisation affect the boundary work’s focus

We observed that two stakeholder characteristics facilitated the SCTs’ boundary work. First, prior familiarity between the stakeholder and the SCT induced the SCT to reach out early to that stakeholder to inform and discuss their vision on the integrated services (Table 3:C). For example, a relationship between SPO members and the football club was already established and enabled the SCT to collaborate and experiment immediately (Table 3:D,E); they organised a focus group with the club’s board members and trainers to discuss ideas. In the following joint activities, the club and the SCT discussed emergent issues with players and with their collaboration and developed learning-by-doing practices (Table 3:D,E). They became familiar faces for trainers, carers, and players, as illustrated by the following quote.

“Every two weeks, I visit the football club during their training sessions and connect with the coaches. Yesterday, a funny thing occurred: a young player called out to me: “Too bad the training is cancelled huh?” So, they recognise me now.” (QUEST21 49 SPO-2)

They, thus, showed progress in their goal to be easily accessible for the inhabitants. This experience empowered SPO to remain outward focused and connect to other trainers, carers, and players, using videos, posters, and training pit visits. As a result, they reached out to other clubs, or own colleagues to inspire them with their progress and success (Table 3:C). In this collaboration between SPO and SCH on the one hand and an external stakeholder on the other hand, they talked about who will act when they signalled a situation in which they needed to collaborate. They agreed about these joint actions based on their knowledge and experience. (Table 3:C,D).

“[…] the housing corporation reached out to us regarding a student renting a studio. This was to prevent the housing issue to escalate. We could jointly coordinate and implement actions quickly, with a positive outcome. […] It’s great that this strengthens collaboration in the chain, and that the housing corporation reaches out to us timely.” (QUEST22 13 SCH-4)

In contrast, the relationship between STR and the multi-problem street’s inhabitants was not sufficiently developed to facilitate the team in devising recognisable reaching out activities. The team started a discussion on who they wanted to be and how they wanted to be recognised by the inhabitants. This internal discussion took up much time (Table 3:A,B), reinforced by the restrictive COVID-19 measures, delaying STR’s reaching out to the inhabitants.

The second stakeholder characteristic was the degree to which the stakeholder was organised as a collectivity (club, school, community centre) and shared the interests with the SCT. SPO and SCH focused on organisations (respectively, sports clubs and schools) sharing the interest of the SCTs, whereas STR focused on a non-organised stakeholder with multiple personal interests (the street) making it hard to approach it as a collectivity. STR’s struggle to work together with them led to inward focused boundary reinforcing (Table 3:A): discussing who they specifically wished to target and how to relate to them as a team. Over time, STR developed ideas, resulting in a flyer for street inhabitants and a webinar for colleagues to reach out to them (Table 3:C). At the end of the project, they developed an integrated team service approach instead of interventions based on the traditional professional roles (Table 3:A). These internal discussions supported their joint appearance in the street.

“We took a cargo bike into the street with coffee, tea, and some [organisation’s name] gadgets. This resulted in more confidence among us to venture into the street as a team and we all talked with the inhabitants. We evaluated this as very positive.” (QUEST22-14 STR-5)

SPO and SCH started from the beginning with a combination of internal discussions and reaching out to others (Table 3:A,C,D). They both initiated a process of learning-by-doing (Table 3:E) to resolve and prevent fragmentation in the social care for the young football players and for students in need of housing support, resulting in a shared integrated approach.

“There is a monthly consultation between us and someone on the club’s board where we discuss the cases.” (QUEST21-11 SPO-3); “The project will be integrated in the club’s policy (QUEST21-7 SPO-1).”

The SCTs showed more outward focused reaching out, collaborating and learning-by-doing activities (Table 3:C,D,E) when they started with or established a firm relationship with members of an organisation. This yielded a positive perceived goal attainment progress. To relate to a non-organised client group seemed to require a strong internal coherence with a clear message towards the clients resulting in more inward focused boundary work (Table 3:A,B). Consequently, the experienced progress in achieving integrated care stayed behind.

### Boundary play encouraged service integration with stakeholders

We observed an unexpected type of boundary work consisting of SCTs’ informal experimenting and learning-by-doing with a stakeholder where existing boundaries were tentatively crossed and possibly rearranged to create more integrated service practices. We labelled these activities as “boundary play”.

Only STR did not show boundary play with a stakeholder but voiced intentions in this direction. The following quote shows their concurrent intention and struggle to get it done. Which led them to give low scores to their own achievements, as also visible in the following reflection:

“The plan to join a street action with the team fell through as three colleagues were absent as well as the inconvenient time because of current COVID-19 regulation. Despite this, it was a nice moment to be present with my colleague, but it felt like a missed opportunity […] I still couldn’t make it happen.” (QUEST 21–51 STR-5)

Instead, STR internally designed a coherent approach through which they wanted to enhance the cohesion among inhabitants, and their youth specifically. They presented this approach to other SCTs and external stakeholders (Table 3:C), which was received favourably: “All SCTs should work in this way”, said a manager. However, as demonstrated above, they did not yet apply their approach in practice.

While, over the 18-months, SPO and SCH engaged in boundary play (Table 3:E) with external stakeholders. The next quotes demonstrate (1) the joint character in creating new collaboration practices, leading (2) to reflections on and updating of action-goals (Figure 2:a).

1. “The last two weeks, we tried to set our joint action-goals as clear as possible to make them useful and resourceful. For example, in terms of ‘early signalling’ carried out by the support team and teachers as well to break through routines and try something new….” (QUEST20-47 SCH-1)
2. “I found it very confronting that I had a conversation with a young player about being suspended from the football club, and only now I started to think how I could help this kid. Perhaps I could have contacted the club to inquire about what had happened and whether there might be an alternative solution instead of a one-year suspension. I find this very unfortunate for an 11-year-old kid. So, suddenly, I see various possibilities.” (QUEST20 50 SPO-2)

SCH and SPO members’ boundary play with a stakeholder was experienced as emergent service integration and fuelled their motivation. The following quote shows such an example of service integration achieved by SCH with an external (OAS) and an internal stakeholder (another SCT) that they reported as impactful for the students.

“The main impact for me is placing young people at [OAS], together with [another SCT] ensuring a fair allocation [of rooms] through easy access and minimising disappointments [through a shared flowchart offering clear routes and criteria]” (QUEST 20-52 SCH-1).

SPO developed a shared approach to signal and help young players with problems that, in monthly meetings with football club representatives, they reflected on and improved.

“We grow more and more toward a knowledge circle of professionals who are connected to youth and sport and wherein our approach can be adapted for the better.” (QUEST20-47 SPO-1).

## Discussion and conclusion

This paper outlined a bottom-up, incremental approach for service integration across SCTs boundaries with stakeholders. Such teams face many (constraining) boundaries in their efforts to integrate services from the care and social domain. We took a boundary work lens to analyse “*how boundary work in SCTs’ practices contribute to service integration in a dynamic multi-stakeholder context*”. The analysis led to four takeaways that contribute to the practice of Social Community Teams in their service integration efforts.

Service integration across the SCT’s and stakeholder’s boundaries requires a range of team boundary work. We identified in this embedded case study five boundary work types which the SCTs displayed and used them to describe how the SCTs worked towards service integration.

The SCTs show boundary reinforcing activities to enhance an inward focused discussion about the way they wanted to collaborate with and been seen by stakeholders. These activities serve as both a stepping-stone and a haven to fall back on when reaching out to stakeholders. Boundary play (Figure 2-4) empowers activities across existing boundaries between the SCT and the stakeholder to integrate service practices, but only when preceded by established boundary spanning activities. The experimental and reflective character of boundary play seemed necessary for integrating the activities, involving communication across boundaries that may encounter a lack of open mindedness [30]. It facilitates both the SCT and its stakeholders to learn and change practices and contributes to achieving service integration practices as a result.

The short-term and long-term action goals impact the SCTs’ boundary work. The first leads to collaboration practices (boundary spanning and play), the second to inward focused discussions (boundary reinforcing). Goal focused reflections support learning-by-doing [31] and supports reconsidering action goals to build new joint and more integrated practices. The action-goal approach is effective for collaboration and exploration by trying new approaches across boundaries when the goals are short-term and action oriented (Figure 2–1). Possibly because they contribute to clarity about the task execution and give direction to reflection and feedback which enhances the goal efficacy in return [32].

Service integration is both a result and a process [33]. Our study shows that the process can accelerate when the SCT creates or maintains a firm relationship with a stakeholder (Figure 2–3). A firm relationship can be seen as social capital: dense, and strong personal ties between the SCT and its stakeholders. These relationships enable the readiness to experiment, and are essential to explore new activities and overcome joint hurdles [34,35,36]. The differences in coping with restricting boundaries (Figure 2–2) resulted in, on the one hand, a continuation of the outward focused joint activities making the restriction a joint effort, and on the other hand, a pause in reaching out, but instead, reinforcing actions clarifying the SCT’s common identity. The nature of the youth’s living places the SCT focus on could play a role. A diffuse target group living in a street (Figure 2–3) makes it difficult to approach as a collectivity to experiment with and develop integrated services.

## Practical and theoretical implications

This study demonstrated the viability of a bottom-up, incremental approach for service integration through long term vision building and members’ short-term stakeholder-directed action-goals, which may also be used by other social community teams. First, when aiming for ground-breaking service integration managers should recognise that a joint experimental and reflection practice contributes to a service integration between stakeholders, and is facilitated by four factors: (1) a team’s bottom-up developed vision for integration, (2) participative short-term action-goal setting by members per stakeholder, (3) inclusion of constraining boundaries into the action-goals as challenges, and (4) the head start of an established relationship with a stakeholder, organised to achieve collective interest. A clear vision is needed for creating a mutual base for joint services, the action-goals as generated from the vision, support collaboration encouraging experimentation. Second, our study shows that these initiatives for service integration need an action-goal focused approach in which the goals of both the SCT and the stakeholder are joined. Third, the incremental, bottom-up approach strengthened the autonomy and motivation of the SCTs by defining its subsequent visions and action-goals, rather than receiving directives imposed by managers or external stakeholders.

Theoretically, we added to the further understanding of types of team boundary work that social community teams perform in their service integration efforts. First, we identified a new boundary work type, coined as ‘boundary play’ that was an important in incrementally realising more integrated services. Second, boundary spanning was demonstrated to work in two complementary ways (a) inside-out focused activities to convey visions and beliefs to stakeholders or simply make your services known, and (b) outside-in focused activities to gain support and resources such as information.

## Limitations and further research

The richness of the data and longitudinal character of this study enhanced the trustworthiness of this study [36]. However, it also has its limitations suggesting areas for further research. One limitation of this study was that it took place during the COVID-19 pandemic which impeded and complicated the SCTs’ actions because of the governmental restrictions to limit contacts to contain the pandemic. The consequence thereof was a limitation of the three SCTs’ action-goal execution to the full extent and consequently, a non-fluent progress in our data collection and data analysis. Although we identified different coping with constraining boundaries, further research can contribute to an additional refinement of the way incorporating the constraints in challenging action-goals can contribute to service integration practices. Furthermore, we identified substantive differences in coping with constraining boundaries, such as the GDPR or the Covid measures, in terms of influence on the SCTs’ boundary activities. However, our research did not consider how and why other boundary types affect the SCTs’ boundary work. Future studies could explore the role of the different boundary types and the interplay between teams’ boundary work. Lastly, it would be worthwhile to explore the concept of boundary play more extensively and in different professional contexts for a better understanding of its role in service integration between teams and stakeholders.
